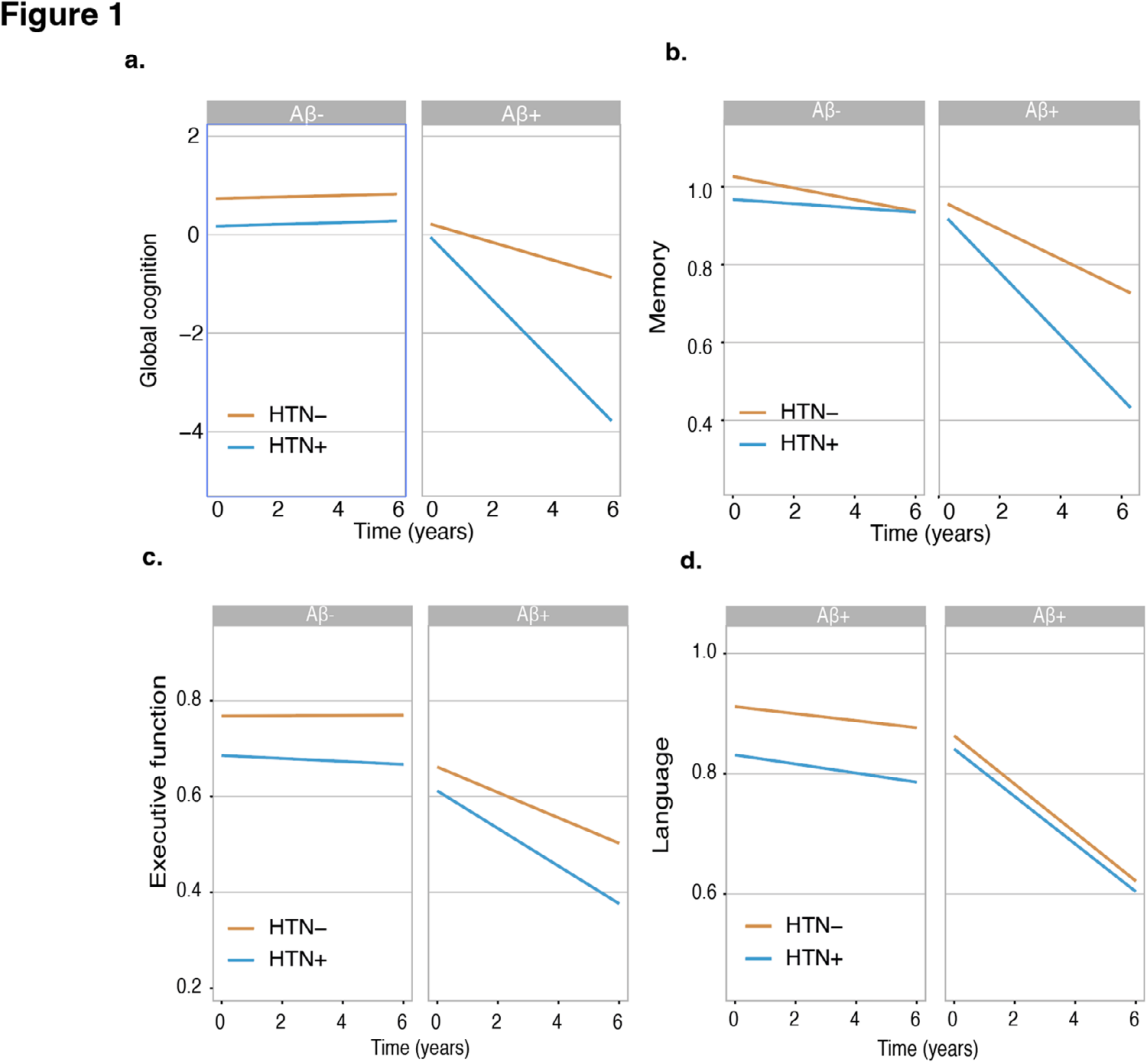# Late‐life hypertension acts together with amyloid‐β pathology to promote memory loss

**DOI:** 10.1002/alz.092284

**Published:** 2025-01-09

**Authors:** Lucas Uglione Da Ros, João Pedro Ferrari‐Souza, Lucas Augusto Hauschild, Marco Antônio de Bastiani, Firoza Z Lussier, Nesrine Rahmouni, Joseph Therriault, Stijn Servaes, Jenna Stevenson, Arthur C. Macedo, Mira Chamoun, Gleb Bezgin, Andrea L. Benedet, Alzheimer's Disease Neuroimaging Initiative (ADNI), Tharick A. Pascoal, Pedro Rosa‐Neto, Eduardo R. Zimmer

**Affiliations:** ^1^ Universidade Federal do Rio Grande do Sul, Porto Alegre Brazil; ^2^ Universidade Federal do Rio Grande do Sul, Porto Alegre, Rio Grande do Sul Brazil; ^3^ Departments of Psychiatry and Neurology, University of Pittsburgh School of Medicine, Pittsburgh, PA USA; ^4^ Federal University of Rio Grande do Sul, Porto Alegre, Rio Grande do Sul Brazil; ^5^ Translational Neuroimaging Laboratory, The McGill University Research Centre for Studies in Aging, Montréal, QC Canada; ^6^ Translational Neuroimaging Laboratory, The McGill University Research Centre for Studies in Aging, Montreal, QC Canada; ^7^ Translational Neuroimaging Laboratory, McGill Centre for Studies in Aging, McGill University, Montréal, QC Canada; ^8^ Laboratory of Neuro Imaging (LONI), University of Southern California, Los Angeles, CA USA; ^9^ University of Pittsburgh School of Medicine, Pittsburgh, PA USA

## Abstract

**Background:**

Midlife hypertension (HTN) is a known risk factor for Alzheimer's disease (AD). However, it remains to be elucidated whether the effect of late‐life HTN is also present. Here, we aimed to assess the associations of late‐life HTN and amyloid‐β pathology (Aβ) with longitudinal changes in global cognition and different domains in cognitively unimpaired (CU) individuals.

**Method:**

We evaluated 475 CU individuals of over 65 years of age from the ADNI cohort, with available baseline medical data and CSF Elecsys biomarkers (Aβ1‐42 and p‐tau181), as well as longitudinal clinical assessments with neuropsychological testing (up to 6 years). We used global cognitive tests, as well as composite scores for memory, executive function and language domains as outcomes. We replicated all the analyses in 162 CU individuals of over 65 years of age from the TRIAD cohort with baseline clinical data and [18F]NAV4694 positron emission tomography (PET) for Aβ, as well as longitudinal clinical assessments with neuropsychological testing (up to 3 years). The presence or absence of HTN was defined based on medical history. In addition, individuals were classified as Aβ+ or Aβ‐ based on CSF p‐tau181/Aβ1‐42 (ADNI) or Aβ‐PET (TRIAD).

**Result:**

Linear mixed‐effects (LME) models showed that HTN and Aβ acted together to promote longitudinal global cognitive decline (ADNI: HTN X Aβ X Time, β = ‐0.44, p = 0.001, Figure 1a; TRIAD: HTN X Aβ X Time, β =‐0.62, p = 0.001). Interestingly, additional analyses demonstrated that HTN and Aβ joint effects were observed on the decline of memory function (ADNI: HTN X Aβ X Time, β = ‐0.05, p = 0.012, Figure 1b; TRIAD: HTN X Aβ X Time, β = ‐0.64, p < 0.001), but not for executive function or and language (Figure 1c, ADNI: p = 0 .6767 and TRIAD: p = 0.797 and Figure 1d, ADNI: p = 0.8961 and TRIAD: p = 0.327, respectively).

**Conclusion:**

In CU individuals at increased risk for AD, late‐life HTN confers increased risk for cognitive decline, particularly in memory function. Further research is needed to understand the mechanisms involved and how treating hypertension in this population may mediate this risk.